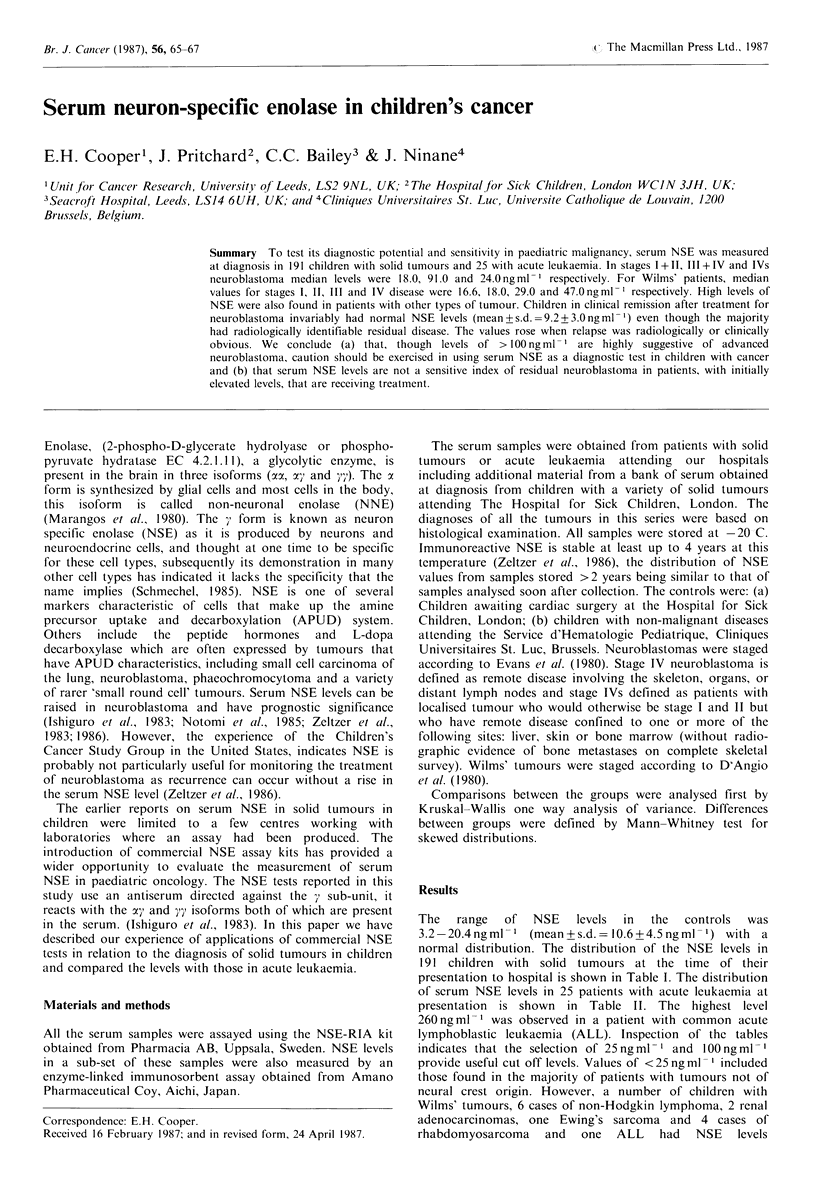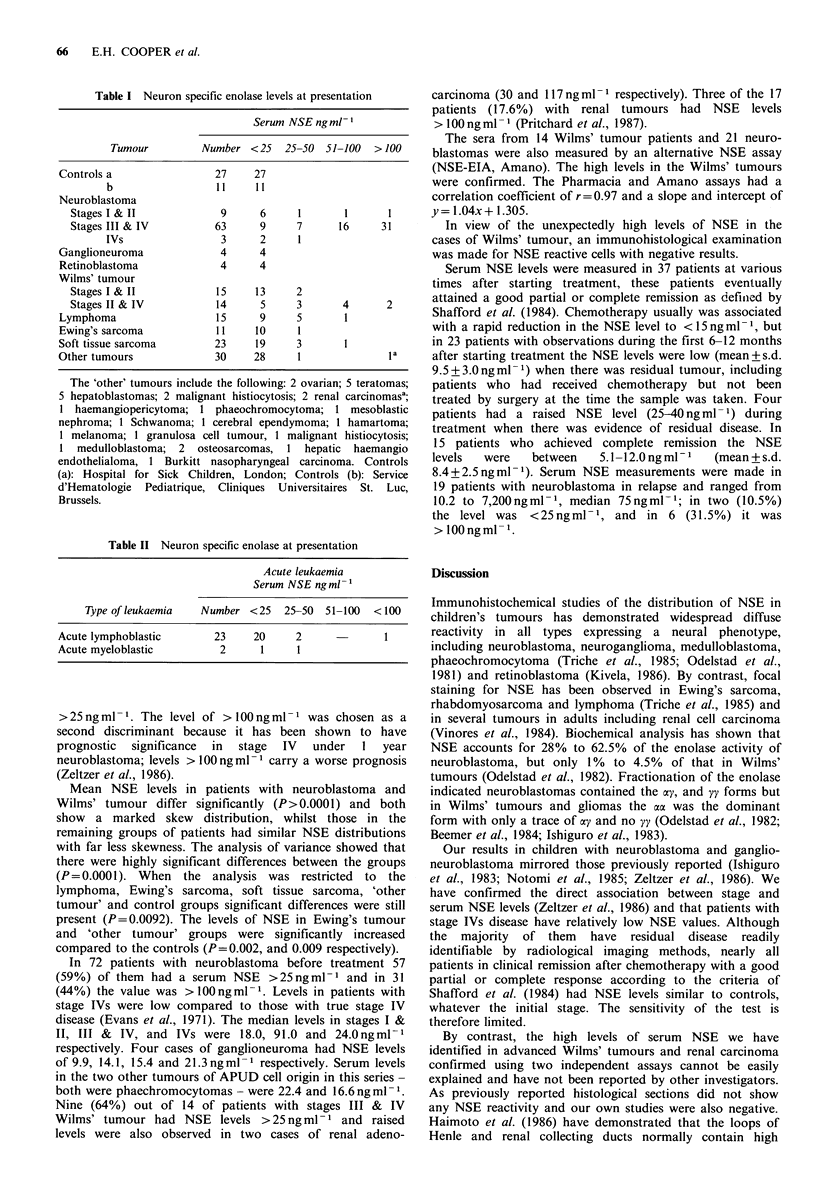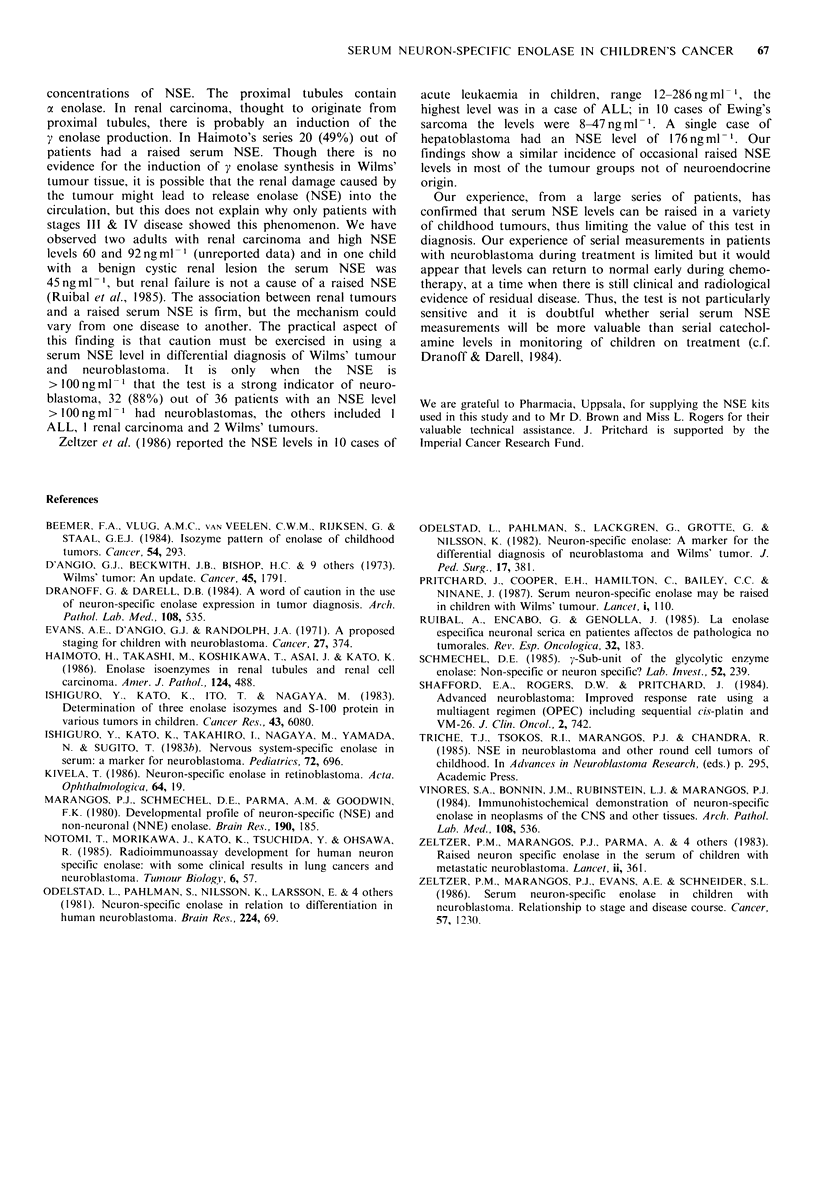# Serum neuron-specific enolase in children's cancer.

**DOI:** 10.1038/bjc.1987.155

**Published:** 1987-07

**Authors:** E. H. Cooper, J. Pritchard, C. C. Bailey, J. Ninane

## Abstract

To test its diagnostic potential and sensitivity in paediatric malignancy, serum NSE was measured at diagnosis in 191 children with solid tumours and 25 with acute leukaemia. In stages I + II, III + IV and IVs neuroblastoma median levels were 18.0, 91.0 and 24.0 ng ml-1 respectively. For Wilms' patients, median values for stages I, II, III and IV disease were 16.6, 18.0, 29.0 and 47.0 ng ml-1 respectively. High levels of NSE were also found in patients with other types of tumour. Children in clinical remission after treatment for neuroblastoma invariably had normal NSE levels (mean +/- s.d. = 9.2 +/- 3.0 ng ml-1) even though the majority had radiologically identifiable residual disease. The values rose when relapse was radiologically or clinically obvious. We conclude (a) that, though levels of greater than 100 ng ml-1 are highly suggestive of advanced neuroblastoma, caution should be exercised in using serum NSE as a diagnostic test in children with cancer and (b) that serum NSE levels are not a sensitive index of residual neuroblastoma in patients, with initially elevated levels, that are receiving treatment.


Br. J. Cancer (1987), 56, 65–67 © The Macmillan Press Ltd., 1987

### Serum neuron-specific enolase in children's cancer

E.H. Cooper[1], J. Pritchard[2], C.C. Bailey[3] & J. Ninane[4]

[1]*Unit for Cancer Research, University of Leeds, LS2 9NL, UK;* [2]*The Hospital for Sick Children, London WC1N 3JH, UK;* [3]*Seacroft Hospital, Leeds, LS14 6UH, UK; and* [4]*Cliniques Universitaires St. Luc, Universite Catholique de Louvain, 1200 Brussels, Belgium.*

**Summary** To test its diagnostic potential and sensitivity in paediatric malignancy, serum NSE was measured at diagnosis in 191 children with solid tumours and 25 with acute leukaemia. In stages I+II, III+IV and IVs neuroblastoma median levels were 18.0, 91.0 and 24.0 ng ml$^{-1}$ respectively. For Wilms' patients, median values for stages I, II, III and IV disease were 16.6, 18.0, 29.0 and 47.0 ng ml$^{-1}$ respectively. High levels of NSE were also found in patients with other types of tumour. Children in clinical remission after treatment for neuroblastoma invariably had normal NSE levels (mean ± s.d. = 9.2 ± 3.0 ng ml$^{-1}$) even though the majority had radiologically identifiable residual disease. The values rose when relapse was radiologically or clinically obvious. We conclude (a) that, though levels of >100 ng ml$^{-1}$ are highly suggestive of advanced neuroblastoma, caution should be exercised in using serum NSE as a diagnostic test in children with cancer and (b) that serum NSE levels are not a sensitive index of residual neuroblastoma in patients, with initially elevated levels, that are receiving treatment.

Enolase, (2-phospho-D-glycerate hydrolyase or phosphopyruvate hydratase EC 4.2.1.11), a glycolytic enzyme, is present in the brain in three isoforms ($\alpha\alpha$, $\alpha\gamma$ and $\gamma\gamma$). The $\alpha$ form is synthesized by glial cells and most cells in the body, this isoform is called non-neuronal enolase (NNE) (Marangos *et al.*, 1980). The $\gamma$ form is known as neuron specific enolase (NSE) as it is produced by neurons and neuroendocrine cells, and thought at one time to be specific for these cell types, subsequently its demonstration in many other cell types has indicated it lacks the specificity that the name implies (Schmechel, 1985). NSE is one of several markers characteristic of cells that make up the amine precursor uptake and decarboxylation (APUD) system. Others include the peptide hormones and L-dopa decarboxylase which are often expressed by tumours that have APUD characteristics, including small cell carcinoma of the lung, neuroblastoma, phaeochromocytoma and a variety of rarer 'small round cell' tumours. Serum NSE levels can be raised in neuroblastoma and have prognostic significance (Ishiguro *et al.*, 1983; Notomi *et al.*, 1985; Zeltzer *et al.*, 1983; 1986). However, the experience of the Children's Cancer Study Group in the United States, indicates NSE is probably not particularly useful for monitoring the treatment of neuroblastoma as recurrence can occur without a rise in the serum NSE level (Zeltzer *et al.*, 1986).

The earlier reports on serum NSE in solid tumours in children were limited to a few centres working with laboratories where an assay had been produced. The introduction of commercial NSE assay kits has provided a wider opportunity to evaluate the measurement of serum NSE in paediatric oncology. The NSE tests reported in this study use an antiserum directed against the $\gamma$ sub-unit, it reacts with the $\alpha\gamma$ and $\gamma\gamma$ isoforms both of which are present in the serum. (Ishiguro *et al.*, 1983). In this paper we have described our experience of applications of commercial NSE tests in relation to the diagnosis of solid tumours in children and compared the levels with those in acute leukaemia.

### Materials and methods

All the serum samples were assayed using the NSE-RIA kit obtained from Pharmacia AB, Uppsala, Sweden. NSE levels in a sub-set of these samples were also measured by an enzyme-linked immunosorbent assay obtained from Amano Pharmaceutical Coy, Aichi, Japan.

Correspondence: E.H. Cooper.
Received 16 February 1987; and in revised form, 24 April 1987.

The serum samples were obtained from patients with solid tumours or acute leukaemia attending our hospitals including additional material from a bank of serum obtained at diagnosis from children with a variety of solid tumours attending The Hospital for Sick Children, London. The diagnoses of all the tumours in this series were based on histological examination. All samples were stored at $-20^\circ$C. Immunoreactive NSE is stable at least up to 4 years at this temperature (Zeltzer *et al.*, 1986), the distribution of NSE values from samples stored >2 years being similar to that of samples analysed soon after collection. The controls were: (a) Children awaiting cardiac surgery at the Hospital for Sick Children, London; (b) children with non-malignant diseases attending the Service d'Hematologie Pediatrique, Cliniques Universitaires St. Luc, Brussels. Neuroblastomas were staged according to Evans *et al.* (1980). Stage IV neuroblastoma is defined as remote disease involving the skeleton, organs, or distant lymph nodes and stage IVs defined as patients with localised tumour who would otherwise be stage I and II but who have remote disease confined to one or more of the following sites: liver, skin or bone marrow (without radiographic evidence of bone metastases on complete skeletal survey). Wilms' tumours were staged according to D'Angio *et al.* (1980).

Comparisons between the groups were analysed first by Kruskal–Wallis one way analysis of variance. Differences between groups were defined by Mann–Whitney test for skewed distributions.

### Results

The range of NSE levels in the controls was 3.2 – 20.4 ng ml$^{-1}$ (mean ± s.d. = 10.6 ± 4.5 ng ml$^{-1}$) with a normal distribution. The distribution of the NSE levels in 191 children with solid tumours at the time of their presentation to hospital is shown in Table I. The distribution of serum NSE levels in 25 patients with acute leukaemia at presentation is shown in Table II. The highest level 260 ng ml$^{-1}$ was observed in a patient with common acute lymphoblastic leukaemia (ALL). Inspection of the tables indicates that the selection of 25 ng ml$^{-1}$ and 100 ng ml$^{-1}$ provide useful cut off levels. Values of <25 ng ml$^{-1}$ included those found in the majority of patients with tumours not of neural crest origin. However, a number of children with Wilms' tumours, 6 cases of non-Hodgkin lymphoma, 2 renal adenocarcinomas, one Ewing's sarcoma and 4 cases of rhabdomyosarcoma and one ALL had NSE levels

**Table I** Neuron specific enolase levels at presentation

| Tumour | Serum NSE ng ml⁻¹ Number | <25 | 25–50 | 51–100 | >100 |
|---|---|---|---|---|---|
| Controls a | 27 | 27 | | | |
| b | 11 | 11 | | | |
| Neuroblastoma | | | | | |
| Stages I & II | 9 | 6 | 1 | 1 | 1 |
| Stages III & IV | 63 | 9 | 7 | 16 | 31 |
| IVs | 3 | 2 | 1 | | |
| Ganglioneuroma | 4 | 4 | | | |
| Retinoblastoma | 4 | 4 | | | |
| Wilms' tumour | | | | | |
| Stages I & II | 15 | 13 | 2 | | |
| Stages II & IV | 14 | 5 | 3 | 4 | 2 |
| Lymphoma | 15 | 9 | 5 | 1 | |
| Ewing's sarcoma | 11 | 10 | 1 | | |
| Soft tissue sarcoma | 23 | 19 | 3 | 1 | |
| Other tumours | 30 | 28 | 1 | | 1[a] |

The 'other' tumours include the following: 2 ovarian; 5 teratomas; 5 hepatoblastomas; 2 malignant histiocytosis; 2 renal carcinomas[a]; 1 haemangiopericytoma; 1 phaeochromocytoma; 1 mesoblastic nephroma; 1 Schwanoma; 1 cerebral ependymoma; 1 hamartoma; 1 melanoma; 1 granulosa cell tumour, 1 malignant histiocytosis; 1 medulloblastoma; 2 osteosarcomas, 1 hepatic haemangio endothelialoma, 1 Burkitt nasopharyngeal carcinoma. Controls (a): Hospital for Sick Children, London; Controls (b): Service d'Hematologie Pediatrique, Cliniques Universitaires St. Luc, Brussels.

**Table II** Neuron specific enolase at presentation

| Type of leukaemia | Acute leukaemia Serum NSE ng ml⁻¹ Number | <25 | 25–50 | 51–100 | <100 |
|---|---|---|---|---|---|
| Acute lymphoblastic | 23 | 20 | 2 | — | 1 |
| Acute myeloblastic | 2 | 1 | 1 | | |

>25 ng ml⁻¹. The level of >100 ng ml⁻¹ was chosen as a second discriminant because it has been shown to have prognostic significance in stage IV under 1 year neuroblastoma; levels >100 ng ml⁻¹ carry a worse prognosis (Zeltzer *et al.*, 1986).

Mean NSE levels in patients with neuroblastoma and Wilms' tumour differ significantly ($P>0.0001$) and both show a marked skew distribution, whilst those in the remaining groups of patients had similar NSE distributions with far less skewness. The analysis of variance showed that there were highly significant differences between the groups ($P=0.0001$). When the analysis was restricted to the lymphoma, Ewing's sarcoma, soft tissue sarcoma, 'other tumour' and control groups significant differences were still present ($P=0.0092$). The levels of NSE in Ewing's tumour and 'other tumour' groups were significantly increased compared to the controls ($P=0.002$, and 0.009 respectively).

In 72 patients with neuroblastoma before treatment 57 (59%) of them had a serum NSE >25 ng ml⁻¹ and in 31 (44%) the value was >100 ng ml⁻¹. Levels in patients with stage IVs were low compared to those with true stage IV disease (Evans *et al.*, 1971). The median levels in stages I & II, III & IV, and IVs were 18.0, 91.0 and 24.0 ng ml⁻¹ respectively. Four cases of ganglioneuroma had NSE levels of 9.9, 14.1, 15.4 and 21.3 ng ml⁻¹ respectively. Serum levels in the two other tumours of APUD cell origin in this series – both were phaechromocytomas – were 22.4 and 16.6 ng ml⁻¹. Nine (64%) out of 14 of patients with stages III & IV Wilms' tumour had NSE levels >25 ng ml⁻¹ and raised levels were also observed in two cases of renal adenocarcinoma (30 and 117 ng ml⁻¹ respectively). Three of the 17 patients (17.6%) with renal tumours had NSE levels >100 ng ml⁻¹ (Pritchard *et al.*, 1987).

The sera from 14 Wilms' tumour patients and 21 neuroblastomas were also measured by an alternative NSE assay (NSE-EIA, Amano). The high levels in the Wilms' tumours were confirmed. The Pharmacia and Amano assays had a correlation coefficient of $r=0.97$ and a slope and intercept of $y=1.04x+1.305$.

In view of the unexpectedly high levels of NSE in the cases of Wilms' tumour, an immunohistological examination was made for NSE reactive cells with negative results.

Serum NSE levels were measured in 37 patients at various times after starting treatment, these patients eventually attained a good partial or complete remission as defined by Shafford *et al.* (1984). Chemotherapy usually was associated with a rapid reduction in the NSE level to <15 ng ml⁻¹, but in 23 patients with observations during the first 6–12 months after starting treatment the NSE levels were low (mean ± s.d. 9.5 ± 3.0 ng ml⁻¹) when there was residual tumour, including patients who had received chemotherapy but not been treated by surgery at the time the sample was taken. Four patients had a raised NSE level (25–40 ng ml⁻¹) during treatment when there was evidence of residual disease. In 15 patients who achieved complete remission the NSE levels were between 5.1–12.0 ng ml⁻¹ (mean ± s.d. 8.4 ± 2.5 ng ml⁻¹). Serum NSE measurements were made in 19 patients with neuroblastoma in relapse and ranged from 10.2 to 7,200 ng ml⁻¹, median 75 ng ml⁻¹; in two (10.5%) the level was <25 ng ml⁻¹, and in 6 (31.5%) it was >100 ng ml⁻¹.

### Discussion

Immunohistochemical studies of the distribution of NSE in children's tumours has demonstrated widespread diffuse reactivity in all types expressing a neural phenotype, including neuroblastoma, neuroganglioma, medulloblastoma, phaeochromocytoma (Triche *et al.*, 1985; Odelstad *et al.*, 1981) and retinoblastoma (Kivela, 1986). By contrast, focal staining for NSE has been observed in Ewing's sarcoma, rhabdomyosarcoma and lymphoma (Triche *et al.*, 1985) and in several tumours in adults including renal cell carcinoma (Vinores *et al.*, 1984). Biochemical analysis has shown that NSE accounts for 28% to 62.5% of the enolase activity of neuroblastoma, but only 1% to 4.5% of that in Wilms' tumours (Odelstad *et al.*, 1982). Fractionation of the enolase indicated neuroblastomas contained the $\alpha\gamma$, and $\gamma\gamma$ forms but in Wilms' tumours and gliomas the $\alpha\alpha$ was the dominant form with only a trace of $\alpha\gamma$ and no $\gamma\gamma$ (Odelstad *et al.*, 1982; Beemer *et al.*, 1984; Ishiguro *et al.*, 1983).

Our results in children with neuroblastoma and ganglioneuroblastoma mirrored those previously reported (Ishiguro *et al.*, 1983; Notomi *et al.*, 1985; Zeltzer *et al.*, 1986). We have confirmed the direct association between stage and serum NSE levels (Zeltzer *et al.*, 1986) and that patients with stage IVs disease have relatively low NSE values. Although the majority of them have residual disease readily identifiable by radiological imaging methods, nearly all patients in clinical remission after chemotherapy with a good partial or complete response according to the criteria of Shafford *et al.* (1984) had NSE levels similar to controls, whatever the initial stage. The sensitivity of the test is therefore limited.

By contrast, the high levels of serum NSE we have identified in advanced Wilms' tumours and renal carcinoma confirmed using two independent assays cannot be easily explained and have not been reported by other investigators. As previously reported histological sections did not show any NSE reactivity and our own studies were also negative. Haimoto *et al.* (1986) have demonstrated that the loops of Henle and renal collecting ducts normally contain high concentrations of NSE. The proximal tubules contain $\alpha$ enolase. In renal carcinoma, thought to originate from proximal tubules, there is probably an induction of the $\gamma$ enolase production. In Haimoto's series 20 (49%) out of patients had a raised serum NSE. Though there is no evidence for the induction of $\gamma$ enolase synthesis in Wilms' tumour tissue, it is possible that the renal damage caused by the tumour might lead to release enolase (NSE) into the circulation, but this does not explain why only patients with stages III & IV disease showed this phenomenon. We have observed two adults with renal carcinoma and high NSE levels 60 and 92 ng ml$^{-1}$ (unreported data) and in one child with a benign cystic renal lesion the serum NSE was 45 ng ml$^{-1}$, but renal failure is not a cause of a raised NSE (Ruibal *et al.*, 1985). The association between renal tumours and a raised serum NSE is firm, but the mechanism could vary from one disease to another. The practical aspect of this finding is that caution must be exercised in using a serum NSE level in differential diagnosis of Wilms' tumour and neuroblastoma. It is only when the NSE is $>100$ ng ml$^{-1}$ that the test is a strong indicator of neuroblastoma, 32 (88%) out of 36 patients with an NSE level $>100$ ng ml$^{-1}$ had neuroblastomas, the others included 1 ALL, 1 renal carcinoma and 2 Wilms' tumours.

Zeltzer *et al.* (1986) reported the NSE levels in 10 cases of acute leukaemia in children, range 12–286 ng ml$^{-1}$, the highest level was in a case of ALL; in 10 cases of Ewing's sarcoma the levels were 8–47 ng ml$^{-1}$. A single case of hepatoblastoma had an NSE level of 176 ng ml$^{-1}$. Our findings show a similar incidence of occasional raised NSE levels in most of the tumour groups not of neuroendocrine origin.

Our experience, from a large series of patients, has confirmed that serum NSE levels can be raised in a variety of childhood tumours, thus limiting the value of this test in diagnosis. Our experience of serial measurements in patients with neuroblastoma during treatment is limited but it would appear that levels can return to normal early during chemotherapy, at a time when there is still clinical and radiological evidence of residual disease. Thus, the test is not particularly sensitive and it is doubtful whether serial serum NSE measurements will be more valuable than serial catecholamine levels in monitoring of children on treatment (c.f. Dranoff & Darell, 1984).

We are grateful to Pharmacia, Uppsala, for supplying the NSE kits used in this study and to Mr D. Brown and Miss L. Rogers for their valuable technical assistance. J. Pritchard is supported by the Imperial Cancer Research Fund.

### References

BEEMER, F.A., VLUG, A.M.C., VAN VEELEN, C.W.M., RIJKSEN, G. & STAAL, G.E.J. (1984). Isozyme pattern of enolase of childhood tumors. *Cancer*, **54**, 293.

D'ANGIO, G.J., BECKWITH, J.B., BISHOP, H.C. & 9 others (1973). Wilms' tumor: An update. *Cancer*, **45**, 1791.

DRANOFF, G. & DARELL, D.B. (1984). A word of caution in the use of neuron-specific enolase expression in tumor diagnosis. *Arch. Pathol. Lab. Med.*, **108**, 535.

EVANS, A.E., D'ANGIO, G.J. & RANDOLPH, J.A. (1971). A proposed staging for children with neuroblastoma. *Cancer*, **27**, 374.

HAIMOTO, H., TAKASHI, M., KOSHIKAWA, T., ASAI, J. & KATO, K. (1986). Enolase isoenzymes in renal tubules and renal cell carcinoma. *Amer. J. Pathol.*, **124**, 488.

ISHIGURO, Y., KATO, K., ITO, T. & NAGAYA, M. (1983). Determination of three enolase isozymes and S-100 protein in various tumors in children. *Cancer Res.*, **43**, 6080.

ISHIGURO, Y., KATO, K., TAKAHIRO, I., NAGAYA, M., YAMADA, N. & SUGITO, T. (1983*b*). Nervous system-specific enolase in serum: a marker for neuroblastoma. *Pediatrics*, **72**, 696.

KIVELA, T. (1986). Neuron-specific enolase in retinoblastoma. *Acta. Ophthalmologica*, **64**, 19.

MARANGOS, P.J., SCHMECHEL, D.E., PARMA, A.M. & GOODWIN, F.K. (1980). Developmental profile of neuron-specific (NSE) and non-neuronal (NNE) enolase. *Brain Res.*, **190**, 185.

NOTOMI, T., MORIKAWA, J., KATO, K., TSUCHIDA, Y. & OHSAWA, R. (1985). Radioimmunoassay development for human neuron specific enolase: with some clinical results in lung cancers and neuroblastoma. *Tumour Biology*, **6**, 57.

ODELSTAD, L., PAHLMAN, S., NILSSON, K., LARSSON, E. & 4 others (1981). Neuron-specific enolase in relation to differentiation in human neuroblastoma. *Brain Res.*, **224**, 69.

ODELSTAD, L., PAHLMAN, S., LACKGREN, G., GROTTE, G. & NILSSON, K. (1982). Neuron-specific enolase: A marker for the differential diagnosis of neuroblastoma and Wilms' tumor. *J. Ped. Surg.*, **17**, 381.

PRITCHARD, J., COOPER, E.H., HAMILTON, C., BAILEY, C.C. & NINANE, J. (1987). Serum neuron-specific enolase may be raised in children with Wilms' tumour. *Lancet*, **i**, 110.

RUIBAL, A., ENCABO, G. & GENOLLA, J. (1985). La enolase especifica neuronal serica en patientes affectos de pathologica no tumorales. *Rev. Esp. Oncologica*, **32**, 183.

SCHMECHEL, D.E. (1985). $\gamma$-Sub-unit of the glycolytic enzyme enolase: Non-specific or neuron specific? *Lab. Invest.*, **52**, 239.

SHAFFORD, E.A., ROGERS, D.W. & PRITCHARD, J. (1984). Advanced neuroblastoma: Improved response rate using a multiagent regimen (OPEC) including sequential *cis*-platin and VM-26. *J. Clin. Oncol.*, **2**, 742.

TRICHE, T.J., TSOKOS, R.I., MARANGOS, P.J. & CHANDRA, R. (1985). NSE in neuroblastoma and other round cell tumors of childhood. In *Advances in Neuroblastoma Research*, (eds.) p. 295, Academic Press.

VINORES, S.A., BONNIN, J.M., RUBINSTEIN, L.J. & MARANGOS, P.J. (1984). Immunohistochemical demonstration of neuron-specific enolase in neoplasms of the CNS and other tissues. *Arch. Pathol. Lab. Med.*, **108**, 536.

ZELTZER, P.M., MARANGOS, P.J., PARMA, A. & 4 others (1983). Raised neuron specific enolase in the serum of children with metastatic neuroblastoma. *Lancet*, **ii**, 361.

ZELTZER, P.M., MARANGOS, P.J., EVANS, A.E. & SCHNEIDER, S.L. (1986). Serum neuron-specific enolase in children with neuroblastoma. Relationship to stage and disease course. *Cancer*, **57**, 1230.